# Nasal Anthropometry: An Assessment Among the Akan and Ewe Ethnic Groups in Ghana

**DOI:** 10.1155/2024/7508791

**Published:** 2024-08-02

**Authors:** Juliet Robertson, Chrissie Stansie Abaidoo, Daniel Kobina Okwan, Joshua Tetteh, Collins Adjei-Antwi, Samuel Bempah, Michael Ampofo

**Affiliations:** Department of Anatomy School of Medicine and Dentistry Kwame Nkrumah University of Science and Technology, Kumasi, Ghana

**Keywords:** ethnic group, leptorrhiny, nose, platyrrhiny, rhinoplasty

## Abstract

The distribution of nasal types has been reported to be influenced by climatic adaptation as the nose is involved in conditioning inhaled air. Previous studies have reported differential nasal types and dimensions among varying populations which is very beneficial in planning for rhinoplasty and in forensic identification. However, there is inadequate data on nasal types and dimensions of the various ethnic groups in the Ghanaian population. Since it is inappropriate to apply nasal dimensions of one ethnic group to another, the current study sought to assess the nasal types and dimensions of Akans and Ewes in the Ghanaian population. Nasal height, nasal length, nasal tip protrusion, morphological nose width, and anatomical nose width were measured from 202 participants (116 Akans and 86 Ewes) aged 18–27 years belonging to the Akan and Ewe ethnic groups. Nasal index was calculated, and the frequencies of the nasal types among the two ethnic groups were determined. Ewe significantly had greater nasal length and nasal tip protrusion than the Akans. For both ethnic groups, sexual dimorphism was observed in morphological nose width and anatomical nose width, with males having greater values than females. The platyrrhine (broad nose) nasal type was predominant among the Akan and Ewe ethnic groups. The average nasal dimensions of the Akan and Ewe ethnic groups for the Ghanaian population have been reported in the present study, which will be useful in rhinoplasty intended for individuals belonging to these ethnic groups and in identification.

## 1. Introduction

The human nose projects externally from the face with its root being continuous with the forehead at the frontonasal suture (nasion) [1]. The nasal cartilaginous skeleton is made up of septal, bilateral greater alar, lateral, lesser alar, and sesamoid cartilages [2]. Anderson, Henneberg, and Norris [3] in a cadaveric study investigating the variability in the anatomy of the nose purported that all the cartilaginous structures especially in the paired cartilages were not symmetrical and differed from one another. Nasal dimensions and the distribution of nasal types have been reported to be influenced by climatic adaptation [4] as the nose is involved in conditioning inhaled air [5, 6]. It has been asserted that dry cold air needs relatively more time and a larger surface area for conditioning, while humid warm air requires relatively less time and a smaller surface area for the conditioning process [6].

The nasal index which determines the nasal type is defined by the mathematical formula, nasal width/nasal height × 100 [4]. The nose is classified as *leptorrhine* (long and narrow) if the nasal index is less than 70, platyrrhine (broad and short) if greater than 85, and mesorrhine (moderate) if between 70 and 85 [4, 7]. Rushikesh and Sushant [4] reported that Caucasians predominantly have leptorrhine type, whereas African Americans and Asians mostly show platyrrhine and mesorrhine types, respectively [8]. Previous studies in Nigeria have reported that the platyrrhine type is predominant among Ijaws, Igbos, and Yoruba ethnic groups in southern Nigeria [9], while the mesorrhine type is common among the Andonis [10].

Nasal indices are among the most important cephalometric parameters useful in interracial and intraracial morphological classification and categorization. Injuries to the nose, malformation of the nose, and seeking a desired nose shape are some of the indications for rhinoplasty [11, 12]. Previous studies on facial anthropometry conducted in the Ghanaian population did not report on the nasal index (nasal shape) existing among the different ethnic groups [13, 14]. Meanwhile, it is inappropriate to apply nasal dimensions of one ethnic group to another [15] either in determination of sex or plastic and oral surgery since geography and nutrition of a particular group of people influence nasal morphology [16]. Accurate nasal measurements fit for a particular ethnic group are required to carry out forensic identification and rhinoplasty. Therefore, the present study sought to determine the nasal types and assess the nasal dimensions of the Akan and Ewe ethnic groups in Ghana.

## 2. Materials and Methods

The present study employed descriptive cross-sectional design. It was conducted from January to December 2022 in the Kwame Nkrumah University of Science and Technology (KNUST), Kumasi. A total of 202 healthy individuals (116 Akans and 86 Ewes) were recruited for the study using a purposive nonrandom sampling technique. The participants were Akans and Ewes aged 18–27 years. The Akans and Ewes are indigenous ethnic groups in Ghana with distinct/varying cultural practices and genetic/ancestry which influence their anatomy including nose shape. Ethical approval was sought from the Committee on Human Research Publication and Ethics, KNUST (reference number: CHRPE/AP/397/21). All participants agreed to join the study, and informed consent was obtained from all individual participants included in the study. Ethnicity and sex of participants were recorded. Participants whose parents and grandparents (both maternal and paternal) did not have interethnic marriages were considered as pure and were included in the study. Also, individuals without nasal abnormalities and injuries and nonpregnant women were included in the study. Individuals with prior facial surgeries, physical indications of endocrine disorders, and oedema were excluded from the present study.

### 2.1. Data Collection

All nasal measurements were taken twice, and the averages in millimetres (mm) were calculated for the determination of nasal index. To avoid interobserver error, all the measurements were taken by the same person and at 10 min apart.

### 2.2. Measurements of Nasal Parameters

**Figure 1:**
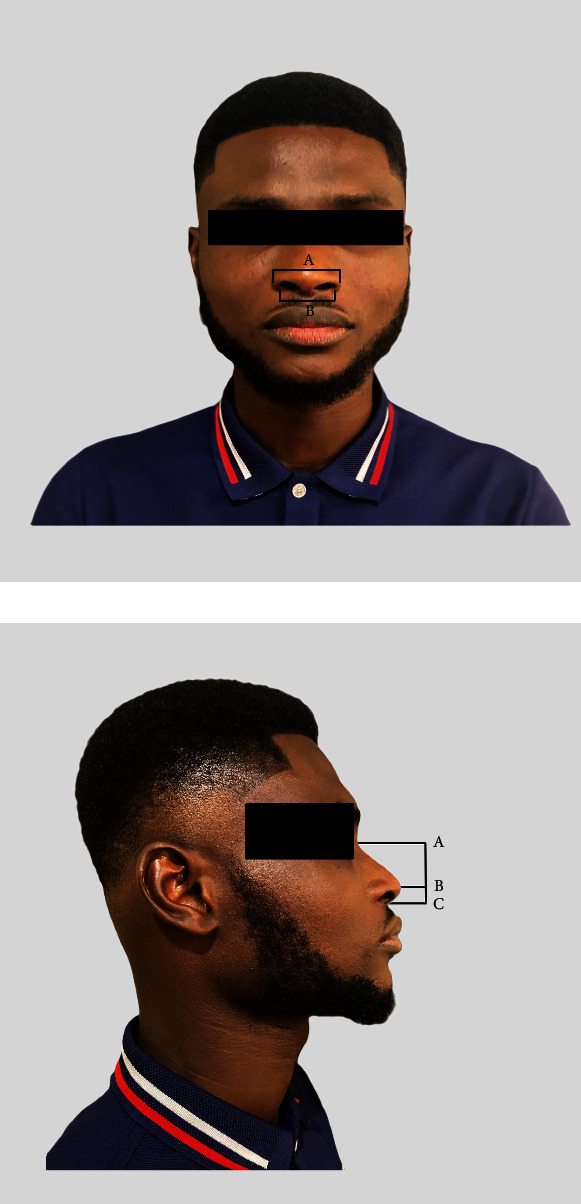
(a) A photograph showing nasal measurements (anterior view): (A) anatomical nose width (ac-ac) and (B) morphological nose width (al-al). (b) A photograph showing nasal measurements (lateral view): (A, B) nasal length (n-pn), (A, C) nasal height (n-sn), and (B, C) nasal tip protrusion (sn-pn).

All nasal measurements were performed with participants in the sitting position: body erect and head up. Using vernier calipers (Shahe Digital Vernier Calipers, 0–150 mm/0.01 mm accuracy), five nasal anthropometric parameters were measured (Figure 1).

1. Nasal height (n-sn): measured as the straight distance from the *nasion* to the *subnasale* in the midsagittal plane.
2. Nasal length (n-pn): measured as the straight distance from the *nasion* to the tip of the nose (pronasale) in the midsagittal plane.
3. Nasal tip protrusion (sn-pn): measured as the straight distance from the *subnasale* to the tip of the nose (pronasale) in the midsagittal plane.
4. Morphological nose width (al-al): measured as the distance between the lateral-most aspects of the left and right *alae nasi*.
5. Anatomical nose width (ac-ac): measured as the distance between the right and left alae curvatures (ac).

### 2.3. Data Analysis

Statistical analyses were carried out using the IBM SPSS Version 25.0, Inc., Chicago, IL, United States. The measurements were expressed in mean ± standard deviation. Normal distribution was tested with the one-sample Kolmogorov–Smirnov test and Shapiro–Wilk normality test. Differences in the means of nasal dimensions across sex and ethnicity were assessed with independent sample *t*-test. The level of statistical significance was determined at *p* < 0.05 or 95% confidence interval.

## 3. Results

Table 1 shows the descriptive statistics of nasal parameters between the Akan and Ewe participants. The Ewes had relatively greater dimensions than the Akan participants. There was no statistically significant difference between nasal parameters of Akans and Ewes except n-pn and sn-pn.

In this study, generally, males recorded greater nasal dimensions than females. For the Akans, sexual dimorphism was observed in sn-pn, al-al, and ac-ac (Table 2). Meanwhile, for the Ewe participants, sexual dimorphism was observed in n-sn, al-al, and ac-ac (Table 3).

Table 4 shows the average nasal indices of males and females of the Akan and Ewe ethnic groups. The nasal index of both sexes within the Akan and Ewe ethnic groups was greater than 80.00 with the males having larger values than the females.

### 3.1. Distribution of Nasal Types Stratified by Ethnicity and Sex

**Figure 2:**
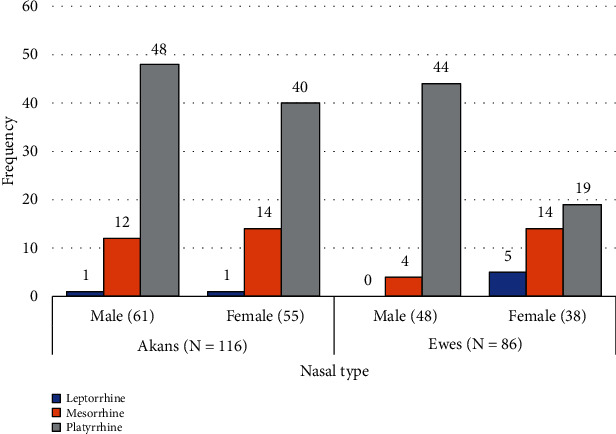
A bar chart showing the nasal index stratified by sex and ethnicity.

Figure 2 is a bar chart showing the frequency of nasal types stratified by sex and ethnicity. Platyrrhine type (broad nose) was the most frequently encountered variation among both sexes within each ethnic group. However, the least recorded type was leptorrhine (fine nose) among both sexes.

## 4. Discussion

From the results of the present study, n-pn and sn-pn differed significantly between the Akan and Ewe ethnic groups (*p* < 0.05). Similarly, previous studies conducted among the Bono and Ewe ethnic groups in Ghana showed a statistically significant difference in n-pn [14] (Table 1). However, Maalman et al. [13] reported a statistically significant difference in all nasal parameters between Dagaaba and Sisaala ethnic groups in Ghana. The results from the present study showed that males had relatively greater nasal dimensions than females for both ethnic groups (Tables 2 and 3). Likewise, a study among ethnic groups in the southern part of Nigeria also reported that males had relatively greater nasal dimensions than females [9]. These findings were in agreement with previous studies conducted among Dagaaba and Sisaala [13], Bono [14], Akans [17], and Central Indian population [18] where there was a statistically significant difference in these parameters between male and female participants. The average nasal height of Akan and Ewe males and females has been reported by the present study (Tables 2 and 3). This implies that in planning for rhinoplasty for individuals belonging to the Akan and Ewe ethnic groups of the Ghanaian population, these normative values can be considered.

Additionally, the average nasal index for both sexes of each ethnic group was greater than 80.00 (Table 4), signifying platyrrhine (broad nose) as the predominant nose type for the participants. Meanwhile, leptorrhine (fine nose) type was the less frequent among the two tribes (Figure 2). Park, Suhk, and Nguyen [8] reported platyrrhine as the predominant nasal type among Africans. Similarly, among the Igbo, Yoruba, and Ijaw in Southern Nigeria, the platyrrhine nasal type was found to be predominant [9]. In a South Indian population, the commonest nasal type was reported to be leptorrhine as nasal index was found to be 67.05 and 64.84 for males and females, respectively [19]. The same study also reported the commonest nasal type of Pradesh population to be mesorrhine. The varying results of the present and previous studies among different populations affirm the use of nose types in identification since it can reveal the ethnic background of an individual. The application of nasal dimensions reported by the current study is of importance in the clinical setting [20]. Additionally, these results confirm the influence of climatic adaptation on nose shape since the nose is involved in conditioning of inhaled air [4]. Elad, Wolf, and Keck [5] asserted that humid warm air requires relatively less time and smaller surface area for conditioning. Hence, individuals chronically exposed to this condition will have short and broad nose as reported by this study.

### 4.1. Limitations

Nasal parameters of other ethnic groups in the country were not investigated, and therefore, findings may not be representative of the entire Ghanaian population. Therefore, nasal parameters of other ethnic groups in the Ghanaian population should be investigated in future studies. Additionally, the use of vernier calipers in measuring nasal parameters was a bit uncomfortable to participants especially because this study was conducted post COVID-19 era where social distancing was prudent. Hence, future studies should consider using indirect methods like photogrammetry in measuring nasal parameters.

## 5. Conclusion

Ewes significantly had greater nasal length and nasal tip protrusion than the Akans. For both ethnic groups, sexual dimorphism was observed in morphological nose width and anatomical nose width, with males having greater values than females. The predominant nasal type among the Akan and Ewe males and females was the platyrrhine followed by the mesorrhine with the leptorrhine having the least frequency among the study population. The present study has provided average nasal dimensions for the Akan and Ewe ethnic groups, which will be useful in rhinoplasty intended for individuals belonging to these ethnic groups and forensic identification.

## Figures and Tables

**Table 1:** Summary of nasal parameters of study participants stratified by ethnicity.

**Parameter**	**Ethnicity**	**N** **=** **202**	**Mean** **±** **SD (mm)**	**p** **value**
Nasal height (n-sn)	Akans	116	44.56 ± 4.84	0.490
	Ewes	86	45.03 ± 4.57	
Nasal length (n-pn)	Akans	116	33.88 ± 5.99	0.005
	Ewes	86	36.32 ± 6.00	
Nasal tip protrusion (sn-pn)	Akans	116	13.11 ± 2.13	≤ 0.001
	Ewes	86	14.42 ± 2.78	
Morphological nose width (al-al)	Akans	116	41.27 ± 5.29	0.429
	Ewes	86	40.68 ± 5.01	
Anatomical nose width (ac-ac)	Akans	116	44.96 ± 4.07	0.626
	Ewes	86	45.27 ± 4.75	

*Note:* Statistically significant difference (*p* < 0.05). Data recorded in mean ± standard deviation (SD).

Abbreviations: ac-ac = alae curvature to alae curvature; al-al = alae to alae; mm = millimetres; *N* = number of participants; n-pn = nasale to pronasale; n-sn = nasion to subnasale; sn-pn = subnasale to pronasale.

**Table 2:** Summary of nasal parameters of Akans stratified by sex.

**Parameter**	**Sex**	**N** **=** **86**	**Mean** **±** **SD (mm)**	**p** **value**
Nasal height (n-sn)	Male	48	45.46 ± 4.93	0.327
	Female	38	44.48 ± 4.08	
Nasal length (n-pn)	Male	48	37.22 ± 6.28	0.117
	Female	38	35.17 ± 5.49	
Nasal tip protrusion (sn-pn)	Male	48	15.24 ± 2.61	0.002
	Female	38	13.38 ± 2.67	
Morphological nose width (al-al)	Male	48	43.39 ± 3.12	≤ 0.001
	Female	38	37.26 ± 4.88	
Anatomical nose width (ac-ac)	Male	48	47.74 ± 4.30	≤ 0.001
	Female	38	42.16 ± 3.25	

*Note:* Statistically significant difference (*p* < 0.05). Data recorded in mean ± standard deviation (SD).

Abbreviations: ac-ac = alae curvature to alae curvature; al-al = alae to alae; mm = millimetres; *N* = number of participants; n-pn = nasale to pronasale; n-sn = nasion to subnasale; sn-pn = subnasale to pronasale.

**Table 3:** Summary of nasal parameters of Ewe ethnic group stratified by sex.

**Parameter**	**Sex**	**N** **=** **116**	**Mean** **±** **SD (mm)**	**p** **value**
Nasal height (n-sn)	Male	61	45.41 ± 5.06	0.046
	Female	55	43.62 ± 4.45	
Nasal length (n-pn)	Male	61	34.78 ± 6.28	0.089
	Female	55	32.88 ± 5.55	
Nasal tip protrusion (sn-pn)	Male	61	13.28 ± 2.44	0.377
	Female	55	12.93 ± 1.74	
Morphological nose width (al-al)	Male	61	42.97 ± 5.41	≤ 0.001
	Female	55	39.38 ± 4.51	
Anatomical nose width (ac-ac)	Male	61	46.12 ± 4.35	0.001
	Female	55	43.68 ± 3.34	

*Note:* Statistically significant difference (*p* < 0.05). Data recorded in mean ± standard deviation (SD).

Abbreviations: ac-ac = alae curvature to alae curvature; al-al = alae to alae; mm = millimetres; *N* = number of participants; n-pn = nasale to pronasale; n-sn = nasion to subnasale; sn-pn = subnasale to pronasale.

**Table 4:** Average nasal index of the study population stratified by sex and tribe.

**Parameter**	**Akans (*N* = 116)** **(mean ± SD)**		**Ewes (*N* = 86)** **(mean ± SD)**	
	**M (61)**	**F (55)**	**M (48)**	**F (38)**
Nasal index	95.53 ± 14.68	91.25 ± 14.52	96.59 ± 13.02	84.12 ± 11.08

Abbreviations: F = females; M = males; *N* = number of participants; SD = standard deviation.

## Data Availability

Data used to support the findings of this study would be made available by the corresponding author upon request.
